# Mitochondrial genome characterization and phylogenetic analysis of *Blastocladiella* sp. (Blastocladiales: Blastocladiaceae)

**DOI:** 10.1080/23802359.2020.1715859

**Published:** 2020-01-24

**Authors:** Xu Wang, Na Liu

**Affiliations:** College of Life Sciences, Henan Agricultural University, Zhengzhou, China

**Keywords:** *Blastocladiella*, Mitochondrial genome, Phylogenetic analysis

## Abstract

In the present study, the complete mitochondrial genome of an early diverging fungus *Blastocladiella* sp. was assembled by the next-generation sequencing. The complete mitochondrial genome of *Blastocladiella* sp. is 33, 800 bp in length and consists of 11,620 (34.38%) adenine, 5,047 (14.93%) cytosine, 6,025 (17.83%) guanosine and 11,108 (32.86%) thymine. The genome contains 19 protein-coding genes, 24 tRNA genes and 2 rRNA genes. Phylogenetic analysis based on the combined mitochondrial gene set showed that *Blastocladiella* sp. has a close relationship with *Allomyces macrogynus* and *Blastocladiella emersonii.*

The genus *Blastocladiella* is a group of saprophytic freshwater fungus, belonging to the phylum Blastocladiomycota (Gomes-Vieira et al. 2018; Luevano-Martinez et al. 2019). *Blastocladiella* spp. are early diverging fungi, located near the base of the fungal phylogenetic tree and close to the separation of fungi, animals and plants (James et al. 2006; Krings et al. 2016). Mitochondrial genome has been widely used to understand the evolution and phylogeny of species due to its rapid evolution rate, single parent inheritance and other characteristics (Li, Chen, et al. 2018). The analysis of mitochondrial genome characterization of *Blastocladiella* species is helpful for us to understand the evolution and origin of the genus *Blastocladiella*.

The specimen *Blastocladiella* sp. was isolated from freshwater in Zhengzhou, Henan, China (113°40′E; 34°23′N) and was stored in Henan Agricultural University (No. Bsp07). Total genomic DNA of the specimen was extracted using a Fungal DNA Kit D3390-00 (Omega Bio-Tek, Norcross, GA, USA), and stored in the sequencing company (BGI Tech, Shenzhen, China). We constructed the sequencing libraries with the extracted genomic DNA using a NEB Next Ultra II DNA Library Prep Kit (NEB, Beijing, China) following the manufacturer’s instructions. Whole genomic sequencing was performed using an Illumina HiSeq 2500 Platform (Illumina, San Diego, CA, USA). The *Blastocladiella* sp. mitochondrial genome was assembled and annotated according to previously described methods (Li, Liao, et al. 2018; Li, Wang, et al. 2018; Li et al. 2019a, 2019b).

The complete mitochondrial genome of *Blastocladiella* sp. is 33, 800 bp in length and consists of 11,620 (34.38%) adenine, 5,047 (14.93%) cytosine, 6,025 (17.83%) guanosine and 11,108 (32.86%) thymine. The genome contains 19 protein-coding genes, 24 tRNA genes and 2 rRNA genes. The *Blastocladiella* sp. mitochondrial genome sequence was submitted to GenBank under the accession number of MN873040.

We used the Bayesian inference (BI) method to create phylogenetic trees based on the combined mitochondrial gene set according to previously described methods (Li, Yang, et al. 2018; Li et al. 2019c). Bayesian analyses were performed with MrBayes v3.2.6 (Ronquist et al. 2012). Phylogenetic analysis showed that *Blastocladiella* sp. exhibited a close relationship with *Allomyces macrogynus* (Paquin and Lang 1996) and *Blastocladiella emersonii* (Tambor et al. 2008) (Figure 1).

**Figure 1.**
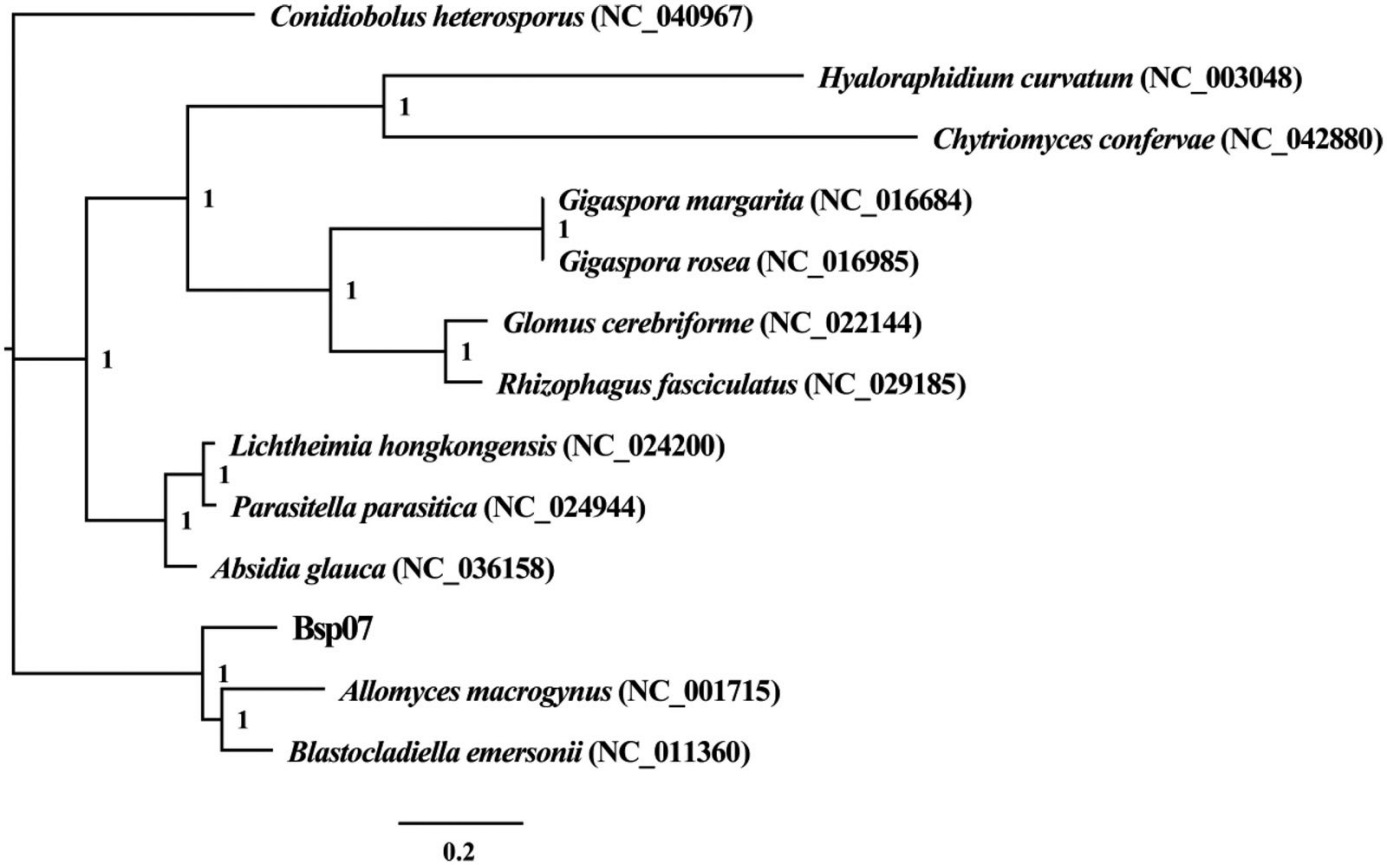
Phylogenetic relationships of 13 species based on Bayesian inference analysis of 14 conserved protein-coding genes. Support values are bayesian posterior probabilities. The brackets after the species name are GenBank accession numbers of species used in the phylogenetic analysis.
